# Germ Cell Development in the Scleractinian Coral *Euphyllia ancora* (Cnidaria, Anthozoa)

**DOI:** 10.1371/journal.pone.0041569

**Published:** 2012-07-27

**Authors:** Shinya Shikina, Chieh-Jhen Chen, Jhe-Yu Liou, Zi-Fan Shao, Yi-Jou Chung, Yan-Horn Lee, Ching-Fong Chang

**Affiliations:** 1 Department of Aquaculture, National Taiwan Ocean University, Keelung, Taiwan; 2 Institute of Oceanography, National Taiwan University, Taipei, Taiwan; 3 Tungkang Biotechnology Research Center, Fisheries Research Institute, Tungkang, Taiwan; 4 Center of Excellence for Marine Bioenvironment and Biotechnology, National Taiwan Ocean University, Keelung, Taiwan; University of Gothenburg, Sweden

## Abstract

Sexual reproduction of scleractinian coral is among the most important means of establishing coral populations. However, thus far, little is known about the mechanisms underlying coral gametogenesis. To better understand coral germ cell development, we performed a histological analysis of gametogenesis in *Euphyllia ancora* and characterized the coral homolog of the *Drosophila* germline marker gene *vasa*. The histological analysis revealed that *E. ancora* gametogenesis occurs in the mesenterial mesoglea between the mesenterial filaments and the retractor muscle bands. The development of germ cells takes approximately one year in females and half a year in males. Staining of tissue sections with an antibody against *E. ancora* Vasa (Eavas) revealed anti-Eavas immunoreactivity in the oogonia, early oocyte, and developing oocyte, but only faint or undetectable reactivity in developing oocytes that were >150 µm in diameters. In males, Eavas could be detected in the spermatogonia and primary spermatocytes but was only faintly detectable in the secondary spermatocytes, spermatids, and sperms. Furthermore, a reverse transcription-polymerase chain reaction analysis and Western blotting analysis of unfertilized mature eggs proved the presence of Eavas transcripts and proteins, suggesting that Eavas may be a maternal factor. Vasa may represent a germ cell marker for corals, and would allow us to distinguish germ cells from somatic cells in coral bodies that have no distinct organs.

## Introduction

The cycles of sexual reproduction and gametogenesis of corals have been studied in various species and sites [1]–[10]. However, thus far, little is known about the mechanisms underlying germ cell development in the coral body. Few studies have aimed to demonstrate the origin of coral germ cells, i.e., germline stem cells. Little is also known about the endocrine factors, such as the hormones and growth factors, that regulate coral germ cell development. Furthermore, it remains unclear whether corals have supporting cells that control the survival, proliferation, and differentiation of germline cells such as the vertebrate Sertoli cells of the testis [11] and follicle cells of the ovary [12]. Because reef-building corals belong to the basal metazoan phylum Cnidaria, which consists of diploblasts that have no true organs [13], 14, investigations for the molecular and cellular endocrine mechanisms underlying germ cell development and somatic germ cell interactions will provide valuable new information, especially to the field of gonadal evolution in metazoans.

As a first step toward addressing these questions, we present here the isolation of a germ cell marker gene for the scleractinian coral *Euphyllia ancora* (Cnidaria, Anthozoa) and histological characterization of *E. ancora* gametogenesis. In Nanwan Bay, southern Taiwan, *E. ancora* inhabits abundantly with forming colonies at depth of approximately 10 m. The polyps of *E.ancora* are extended day and night and are large enough in size (approximately ∼3 cm in diameters, ∼6 cm in heights in each polyp). The size of polyps is suitable as the materials for the studies of molecular biology (e.g. RNA, DNA, and protein), endocrinology (e.g. tissue extractions), and histology. In sexual reproduction, *E. ancora* produce only male or female gametes within each colony. In late spring, they release gametes in synchrony for external fertilization during a brief periods 1∼2 hours after sunset. We have previously shown that *E. ancora* displayed elevated sex steroid levels, aromatase activity, and immunoreactive gonadotropin-releasing hormone as approaching to the spawning season [15]–[17], raising a possibility that these molecules may play an important role in the control of coral germ cell development and maturation as in vertebrate species [18]. Therefore, the information about germ cell development of *E. ancora* would be valuable for the studies on endocrine system of reproduction in the coral.

To understand the mechanism by which hormones, growth factors and cell-cell interactions mediate intercellular communication in corals, the precise assignment of cell types is required. Additionally, because corals have no true organs [14], it is difficult to unambiguously distinguish germ cells from somatic cells. The use of molecular markers to identify germ cells during their gametogenesis is generally accepted as one of the most reliable ways. For instance, using evolutionarily-conserved genes expressing in germ cells as molecular markers has proven useful in identifying germ cells and investigating their behaviors in vertebrates and invertebrates [19]–[22]. However, thus far, only a few germ cell marker genes (related to late germ cell markers) have been reported in corals [23], [24].

Here, we focus on the coral equivalent of the *Drosophila vasa* gene as a germ cell marker for corals. Vasa is a member of an ATP-dependent RNA helicase family of DEAD-box proteins and is capable of unwinding double-stranded RNA loops to promote the translation of germline-specific target mRNAs [25]–[27]. The *vasa* transcripts and protein have been used as universal germ cell markers in a variety of metazoans [22] including cnidarians [28]–[30]. Also, it was shown that vasa gene products are maternally inherited and acted as one of the germ cell determinants in several metazoans [31]–[33].

We reported here the cloning of the full-length coral *vasa* gene and the generation of an anti-Vasa antibody. We also provided a histological analysis of gametogenesis in *E. ancora* and immunohistochemical characterization of Vasa-expressing cells and Vasa intracellular localization. We showed that Vasa is produced in the germ cells of both males and females. Using the anti-Vasa antibody, we were clearly able to observe the early germ cells such as oogonia, early oocytes, and early spermatogonia that have not been intensively described in previous researches, suggesting that vasa may be a useful marker for germ cell development in corals.

## Results

### Reproduction of *Euphyllia ancora*

**Figure 1:**
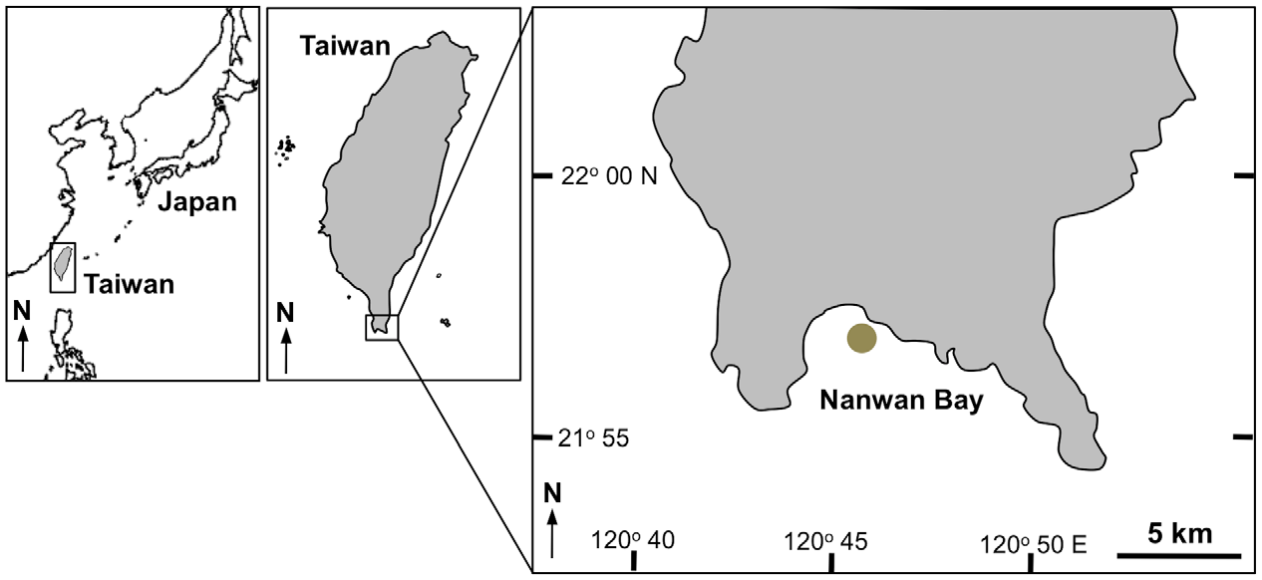
Maps of the sampling location. The maps are showing the sampling location in Nanwan Bay at the southern coast of Taiwan.

**Figure 2:**
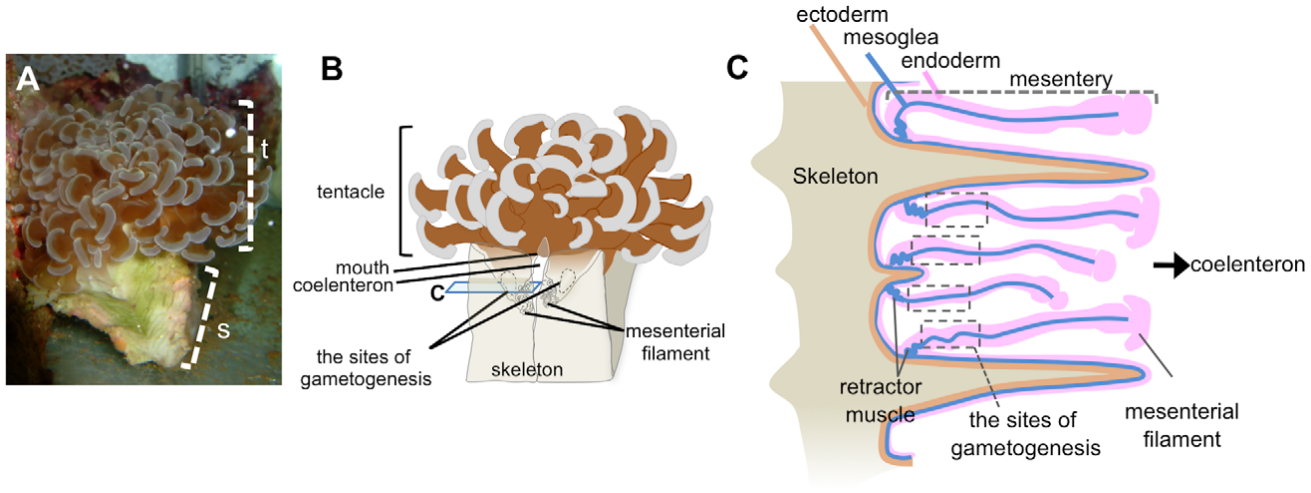
Photographic and schematic representation of the body structure of *E. ancora*. A. *E. ancora* in an aquarium. The brown color in the tentacles is from *Symbiodinium sp*. t, tentacle; s, skeleton. B. Schematic of *E. ancora* body structure. C. Schematic of *E. ancora* horizontally dissected at the point indicated in B. The sites of gametogenesis are located between the retractor muscle and mesentery filament in the mesentery.

**Figure 3:**
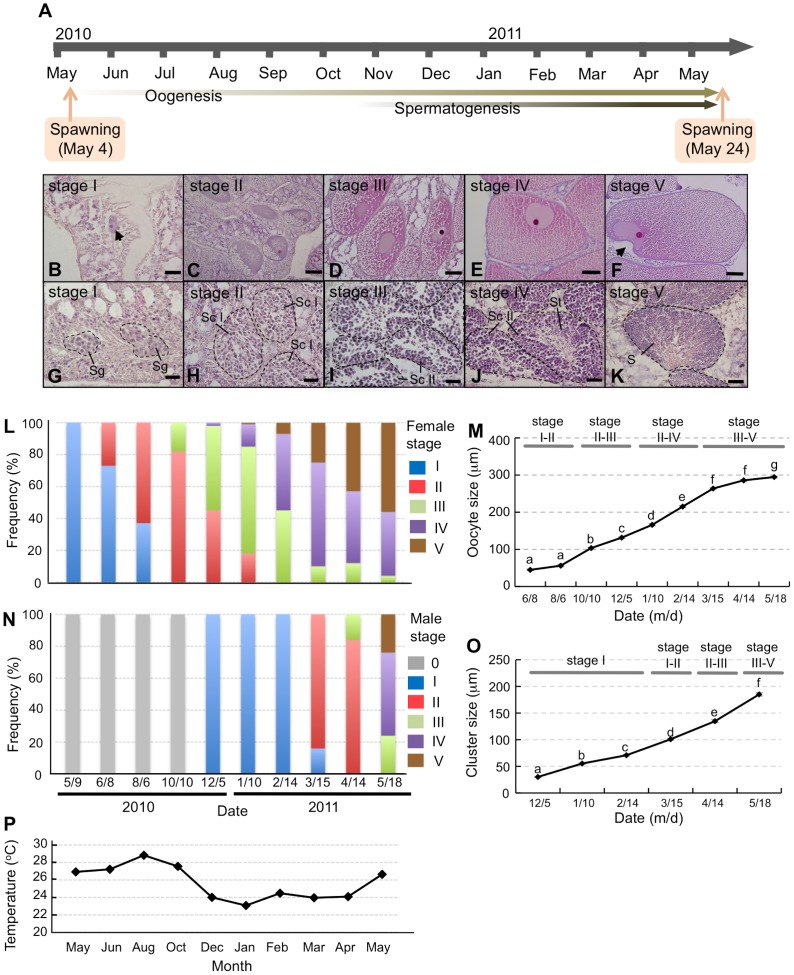
Reproductive cycle of *E. ancora* in both female and male according to histological analysis by H & E staining. **A**. Periods of male and female gametogenesis and dates of mass spawning between May 2010 and May 2011. **B–F**. Stages of oogenesis. **B**. Stage I. The arrow points to an oocyte. **C**, stage II; **D**, stage III; **E**, stage IV; and **F**, stage V. The arrow indicates a germinal vesicle that appears ‘U’-shaped due to the indentation of the plasma membrane. **G–K**. Stages of spermatogenesis. **G**. stage I. **H**, stage II; **I**, stage III; **J**, stage IV; and **K**, stage V. The broken lines indicate male germ cell clusters. Sg, spermatogonia; Sc I, primary spermatocytes; Sc II, secondary spermatocytes; St, spermatids; S, sperms. The scale bars represent 20 µm in panels **B** and **G–K** and 50 µm in panels **C–F**. The stage classifications are referred to Table 1. Gametogenesis frequency distribution for male and female *E. ancora* sampled between May 2010 and May 2011. **L**. The frequency distribution of oogenic stages among female corals (n = 3 colonies). Stage classification was performed according to the criteria provided in Table 1. **M**. Temporal changes in mean diameter of oocytes. The data are reported as the mean ± SEM (June 8, n = 30 eggs; August 6, n = 64 eggs; October 10, n = 88 eggs; December 5, n = 298 eggs; January 10, n = 236 eggs; February 14, n = 306 eggs; March 15, n = 782 eggs; April 14, n = 519 eggs; May 18, n = 441 eggs). Significant differences (*P*<0.05) are indicated with the different lower case letters. **N**. The frequency distribution of spermatogenic stages among male corals (n = 3 colonies). Stage classification was performed according to the criteria provided in Table 1. **O**. Temporal changes in mean diameter of male germ cell cluster. The data are expressed as the mean ± SEM (Cluster number: December 5, n = 104; January 10, n = 102; February 14, n = 100; March 15, n = 102; April 14, n = 103; May 18, n = 111). Significant differences (*P*<0.05) are indicated with the different lower case letters. **P**. Monthly mean temperatures of the sampling sites.

*E. ancora* were collected in Nanwan Bay at southern Taiwan (Fig. 1). They were gonochorists, and we observed the release of *E. ancora* gametes during a brief annual spawning period on May 4, 2010. To understand when and where gametogenesis occurs in *E. ancora* (Fig. 2 A) coral bodies, we labeled the colonies and sampled at different times from May 9 to the next spawning period, i.e., May 24, 2011, and examined the tissue histologically. From these analyses, we found that cells undergoing gametogenesis were localized in the mesenterial mesoglea between the mesenterial filaments and retractor muscles in the endoderm (Fig. 2 B and C). The classification of gametogenic stage according to cell size and morphology (Table 1) revealed that the development of female germ cells to mature oocytes takes approximately one year, whereas the development of male germ cells into mature sperms takes approximately half a year (Fig. 3 A).

**Table 1:** Criteria for classification of stages in gonadal development, as observed in histological sections.

Stage	Male	Female
0	No spermatogonial clusters in mesentery.	No distinct oocytes in mesentery.
I	Formation of spermatogonial clusters in mesentery.	Oocytes of 20–50 µm in diameters.
II	Cluster boundaries distinct. Spermatogonia or primary spermatocytes with small nuclei.	Oocytes of 51–125 µm in diameters.
III	Larger clusters of secondary spermatocytes having condensed nuclei and small cytoplasm.	Oocytes of 126–200 µm in diameters.
IV	Spermatocytes undergoing second division of meiosis and distinct spermatids appear with long tail.	Oocytes of 201–275 µm in diameters.
V	Sperms.	Oocytes of >276 µm in diameters.

### Histological analysis of oogenesis by H & E staining

Just after spawning in 2010, which was May for the females, it was difficult to unambiguously distinguish oogonia from somatic cells under our observation (stage 0). The earliest stage of female germ cells that could be distinguished was oocytes of 20–50 µm in diameters, located along the mesenterial mesoglea of samples collected after the spawning period in May and June (stage I, Fig. 3 B and L). The number of recognizable oocytes increased between August and October, and most of the oocytes at this stage were located within the mesoglea (stage II, Fig. 3 C and L). The later stage oocytes demonstrated both an accumulation of cytoplasmic yolk and an increase in size. The mean oocytes diameters were significantly increased in the samples collected during August to March (Fig. 3 M, *P*<0.05) and reached stages III–V (Fig. 3 D–F and L). Most of the oocytes collected between April and May had reached a full size of ∼250–300 µm in diameters (Fig. 3 M) and demonstrated a semicircular or ‘U’-shaped germinal vesicle caused by indentation of the plasma membrane (stage V, Fig. 3 F). The frequency distributions of oogenic stage were similar among the colonies that we observed (data not shown).

### Histological analysis of spermatogenesis by H & E staining

Between June and October for the males, it was difficult to unambiguously distinguish the spermatogonia from somatic cells under our observation (stage 0, Fig. 3 N). The earliest stage of male germ cells that we identified was spermatogonia resided in clusters alongside the mesenterial mesoglea in corals sampled in December (stage I, Fig. 3 G and N). The most of spermatogonia entered into the mesoglea, proliferated, and differentiated into primary spermatocytes by March (stage II, Fig. 3 H and N). By a few weeks before the next spawning period, most of the primary spermatocytes had undergone meiotic division to form secondary spermatocytes, spermatids, and sperms (stages III–V, Fig. 3 I–K and N). During March to May, we found asynchrony in spermatogenic stages within identical polyp, and also among colonies (data not shown). We also found that spermatogenic development involved an increase in size of germ-cell clusters (Fig. 3 O). The mean diameters of germ-cell cluster were significantly increased in the samples collected from December (30±10.8 µm) to May (185±32.2 µm) (Fig. 3 O).

### Isolation, characterization, and phylogenetic analysis of *E. ancora* vasa cDNA

**Figure 4:**
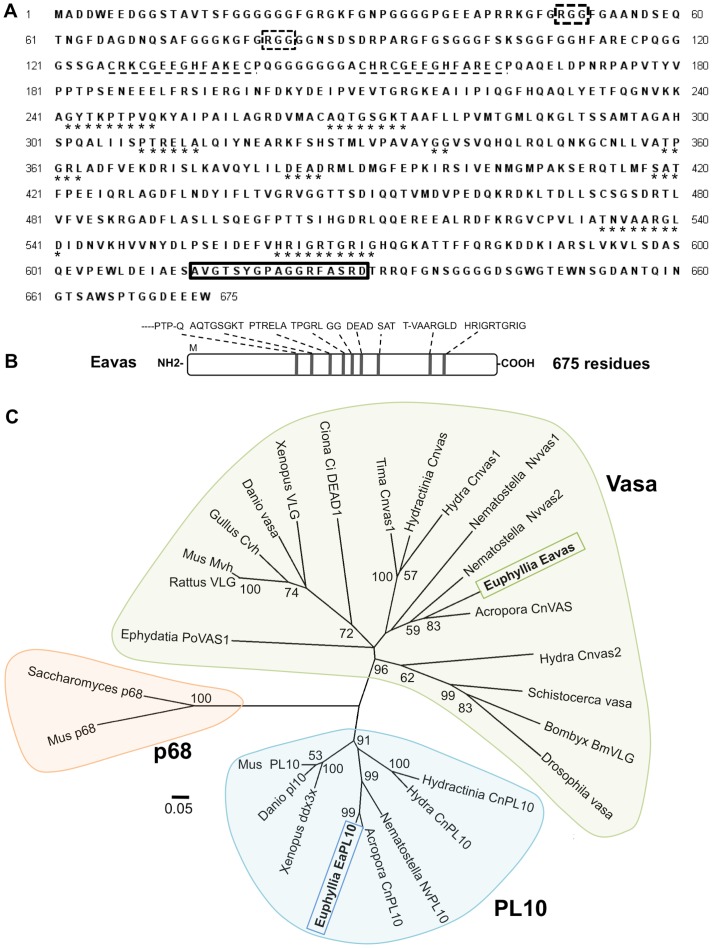
The deduced protein sequence of Eavas and schematic of the Eavas domain structure. **A**. The deduced Eavas amino acid sequence. *, motifs conserved among DEAD-box protein family members; *black dotted line*, CCHC zinc fingers; *black box with dotted line*, RGG motif; *black box*, location of the antigen sequence used for antibody production. **B**. Schematic figure depicting the Eavas domain structure and regions that are highly homologous among the Vasa subfamilies. Vertical lines mark the positions of the nine conserved DEAD-box motifs. **C**. A phylogenetic tree comparing the amino acid sequences of Vasa- and PL10-related proteins from various taxa. The region used for this analysis corresponds to the sequence AFLLPV…LDEA (from 276 to 385 residues of Eavas), where are available and comparable region among various taxa. The sequences were aligned by a multiple sequence alignment using MUSCLE. The phylogenetic tree was constructed using the neighbor-joining method. The number at each node represents the bootstrap probability (%); the branches shown correspond to values of 50% and higher. The names and corresponding GenBank accession numbers of the proteins analyzed are as follows: Acropora CnVas (*Acropora digitifera*, BAB13683), Euphyllia Eavas (*Euphyllia ancora*, JQ968407), Nematostella Nvvas1 (*Nematostella vectensis*, AAW29073), Nematostella Nvvas2 (*Nematostella vectensis*, AAW29074), Tima Cnvas1 (*Tima Formosa*, BAB13687), Hydractinia Cnvas (*Hydractinia echinata*, BAB13686), Hydra Cnvas1 (*Hydra vulgaris*, BAB13307), Hydra Cnvas2 (*Hydra vulgaris*, BAB13308), Ephydatia PoVAS1 (*Ephydatia fluviatilis*, BAB13310), Ciona Ci DEAD1 (*Ciona intestinalis*, BAA36710), Xenopus XVLG1 (*Xenopus laevis*, NP_001081728), Danio vasa (*Danio rerio*, AAI29276), Gallus Cvh (*Gallus gallus*, BAB12337), Rattus VLG (*Rattus sp*., AAB33364), Mus Mvh (*Mus musculus*, BAA03584), Schistocerca vasa-like (*Schistocerca gregaria*, AF510054), Drosophila vasa (*Drosophila melanogaster*, NP_723899), Bombyx BmVLG (*Bombyx mori*, BAA19572), Acropora CnPL10 (*Acropora digitifera*, BAB13676), Euphyllia EaPL10 (*Euphyllia ancora*, JQ968406), Nematostella NvPL10 (*Nematostella vectensis*, AAW29072), Hydractinia CnPL10 *(Hydractinia echinata*, BAB13679), Hydra CnPL10 (*Hydra vulgaris*, BAB13306), Ephydatia PoPL10 (*Ephydatia fluviatilis*, BAB13309), Danio pl10 (*Danio rerio*, NP_571016), Xenopus ddx3x (*Xenopus laevis*, NP_001080283), Mus PL10 (*Mus musculus*, AAA39942), Mus p68 (*Mus musculus*, CAA46581), and Saccharomyces p68 (*Saccharomyces cerevisiae*, CAA36874).

To determine whether the vasa gene product could be used as a germ cell marker in *E. ancora*, we cloned the full length *E. ancora vasa* (*Eavas*) cDNA. The *Eavas* cDNA was 2,526 bp in length and contained an open reading frame of 2,025 bp corresponding to 675 amino acid residues, a 5′ untranslated region (UTR) of 220 bp, and a 3′UTR of 278 bp including a poly A tail (*Eavas*; GenBank accession number JQ968407). The deduced amino acid sequence of Eavas contains the nine conserved domains characteristic of other Vasa-like proteins (Fig. 4 A and B): the Q-motif (XXXXPTPXQ), ATPase motifs (AXXGXGKT, DEAD), the motifs involved in ATP binding and cleavage (PTRELA, GG, TPGRL), the RNA unwinding motifs (SAT, HRIGRTGRIG), and the helicase C domain (TXVAARGXD). Additionally, in N-terminus region, Eavas contains RGG motifs and two CCHC-type zinc fingers (C-X_2_-C-X_4_-H-X_4_-C) (Fig. 4 A). Analysis of the phylogenetic relationship between DEAD-box protein family members showed that Eavas clusters with other cnidarian Vasa proteins and is not the closely related to PL10 proteins (Fig. 4 C).

### RT-PCR and Western blot analysis of *E. ancora* vasa

**Figure 5:**
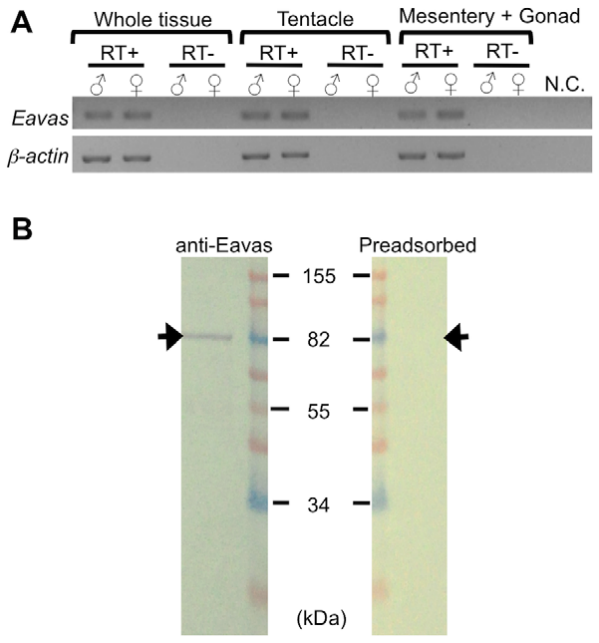
Tissue distribution of *Eavas* mRNAs and characterization of the anti-Eavas antibody. **A**. The tissue distribution of *Eavas* transcripts was determined by semiquantitative RT-PCR analysis of male and female coral samples collected in Februay 2011. The samples examined include whole tissue, isolated tentacles, and the mesentery, the latter of which encompasses the gonad. β-Actin was used as the internal control. Reactions lacking either the reverse transcriptase (RT−) or template (N.C.) were included as negative controls for each set of reactions. **B**. Western blotting analysis of the anti-Eavas antibody. Protein extracts prepared from a Februay 2011 male sample (12.5 µg) were separated by SDS-PAGE. After transferring to a nitrocellulose membrane, the proteins were immunoblotted with the anti-Eavas antibody (anti-Eavas) or the anti-Eavas antibody preadsorbed with the peptide antigen (preadsorbed). The molecular weight markers are shown in the middle.

RT-PCR of cDNAs from isolated tentacles and mesentery tissue (where the germ-cell developing regions are located) showed that the *Eavas* transcripts were present in both the tentacles and mesentery tissue at a similar level in both sexes (Fig. 5 A). The control reactions lacking either the reverse transcriptase or template cDNA did not yield an amplified product (Fig. 5 A). Next, we developed an anti-Eavas antibody for the *in situ* characterization of coral germ cells. Western blotting with the anti-Eavas antibody revealed a single immunoreactive band of approximately 82 kDa (Fig. 5 B) that could be eliminated by pre-adsorption of the antibody with the peptide antigen (Fig. 5 B).

### Immunoreactive *E. ancora* Vasa-positive cells in the corals

**Figure 6:**
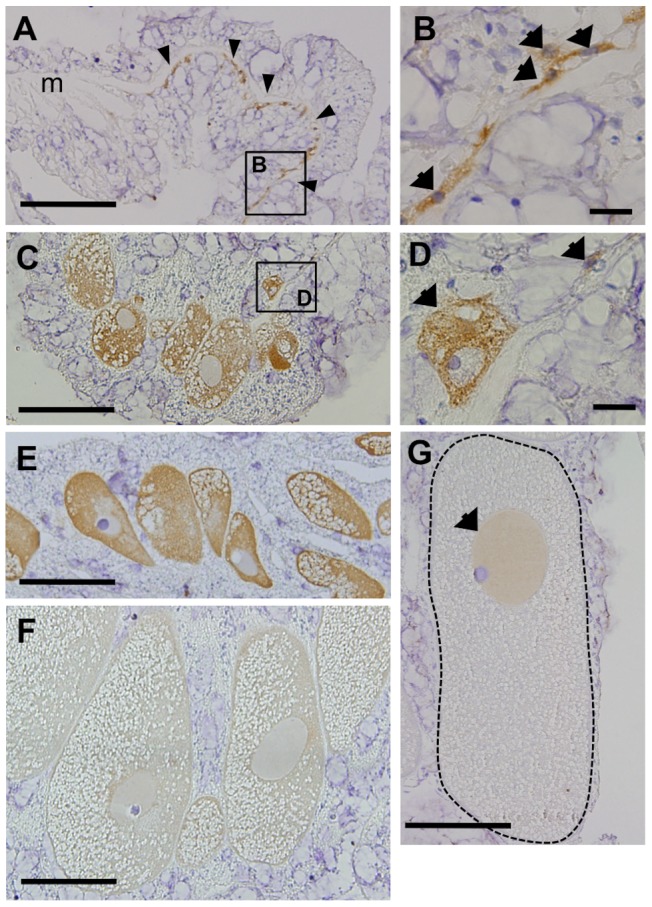
Characterization of anti-Eavas immunoreactivity in female colonies. Immunohistochemical analysis with the anti-Eavas antibody was performed to examine Eavas immunoreactivity (irEavas) in cells at different developmental stages of oogenesis. **A**. irEavas-positive oogonia located along the mesoglea (arrowheads) of samples harvested in May, just 5 days after spawning. m, mesoglea. **B**. A higher magnification view of the inset shown in **A**. Arrows indicate oogonia that display irEavas staining. **C**. irEavas-positive oocytes in the mesentery of corals that were sampled in August (stages I–II). **D**. A higher magnification view of the inset shown in **C**. The arrows indicate oocytes that display irEavas staining. **E**. irEavas-positive oocytes in the mesentery of coral samples collected in October (stage II). **F**. irEavas-positive oocytes from a February coral sample (stages III–IV). **G**. An oocyte (dotted line) from a coral sample collected in April (stage V). The arrows indicate irEavas-positive nuclei. The scale bars correspond to 100 µm in panels **A**, **C**, and **E–G** and 10 µm in panels **B** and **D**.

Immunohistochemical analysis of female corals revealed that Eavas was present in small number of putative oogonia in the samples collected 5 days after spawning (stage 0, May 9, 2010). They occurred alongside the mesenterial mesoglea with amoeboid shape (Fig. 6 A and B). Their nuclei sizes were approximately 4 µm (Fig. 6 B). Also, Eavas was present in both the early (stages I–II, Fig. 6 C and D) and developing (stages II–III, Fig. 6 E) oocytes. The immunoreactivity observed in the oocyte cytoplasm was much fainter in later stage oocytes that exceeded 150 µm in diameters (Fig. 6 F). The immunoreactivity was observed in the nucleus of mature oocytes collected between April and May (stages IV–V, Fig. 6 G).

**Figure 7:**
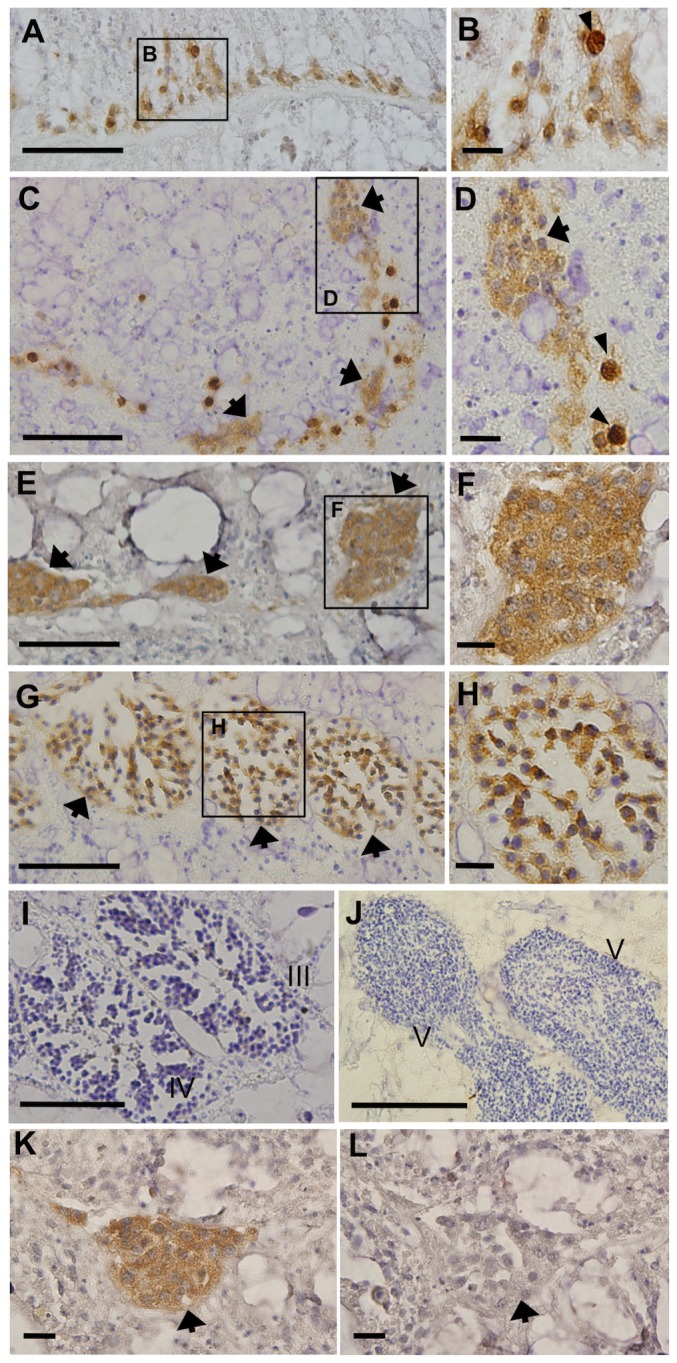
Characterization of anti-Eavas immunoreactivity in male colonies. Immunohistochemical analysis with the anti-Eavas antibody was conducted to examine Eavas immunoreactivity (irEavas) in cells at different stages of spermatogenesis. **A**. irEavas-positive spermatogonia located along the mesenterial mesoglea of a coral sample harvested in August (stage 0). **B**. A higher magnification view of the inset shown in **A**. Arrowheads indicate spermatogonia that display darker Eavas staining than others. **C**. Spermatogonia located along the mesenterial mesoglea of a coral sample harvested in October (late stage 0). The arrows indicate small spermatogonial clusters. **D**. A higher magnification view of the inset shown in **C**. The arrow indicates a spermatogonial cluster. The arrowheads indicate spermatogonia that exhibit darker irEavas staining compared with other spermatogonia. **E**. irEavas-positive spermatogonia located in the mesentery of a coral sampled in December (stage I). The arrows indicate spermatogonial clusters. **F**. A higher magnification view of the inset shown in **E**. **G**. irEavas stained spermatocytes located in the mesentery of a coral sampled in March (stage II). The arrows indicate the clusters within the mesoglea. **H**. A higher magnification view of the inset shown in **G**. **I**. Immunohistochemical analysis with the anti-Eavas antibody to examine Eavas immunoreactivity in stage III and IV male germ cells that were collected in April. **J**. Immunohistochemical analysis of stage V male germ cells collected in May. III, stage III; IV, stage IV; V, stage V. **K**. Immunohistochemical characterization of the anti-Eavas antibody using stage I male germ cells. **L**. The anti-Eavas antibody was preadsorbed with the peptide antigen. The preadsorbed control sample was nearly devoid of immunoreactivity. The arrow points to a cluster of male germ cells. The scale bars correspond to 50 µm in panels **A**, **C**, **E**, **G**, **I** and **J**, and 10 µm in panels **B**, **D**, **F**, **H**, **K** and **L**.

Immunohistochemical analysis of male corals revealed that Eavas was present in unclustered putative spermatogonia in June-August (stage 0, Fig. 7 A and B). They occurred alongside the mesenterial mesoglea with amoeboid shape (Fig. 7 A and B). Their nuclei sizes were approximately 4–6 µm (Fig. 7 B). Also, Eavas was present in spermatogonia captured at the onset of cluster formation (late stage 0, October; Fig. 7 C and D). At this stage, we observed that a part of early male germ cells exhibited darker immunoreactive Eavas signals than others (Fig. 7 A–D). Eavas was also present in the clusters of spermatogonia (stage I, December; Fig. 7 E and F) and primary spermatocytes (stage II, March; Fig. 7 G and H). Eavas staining was faint or almost undetectable in the secondary spermatocytes, spermatids, and mature sperms collected between April and May (stages III–V, Fig. 7 I and J). The signal generated with the anti-Eavas antibody was completely abolished by co-incubation of the antibody with the peptide antigen during the staining procedure (Fig. 7 K and L).

**Figure 8:**
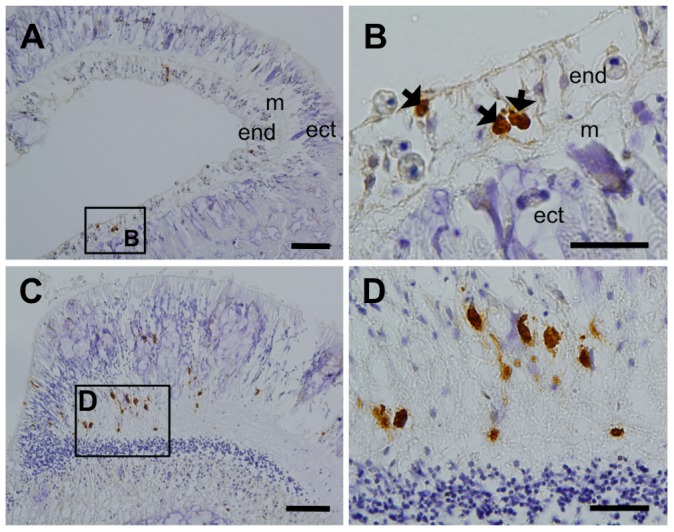
Characterization of anti-Eavas immunoreactivity in tentacle and mesenterial filament. **A**. irEavas-positive cells in the tentacle region. **B**. Higher magnification views of the insets shown in **A**. The arrows indicate irEavas-positive cells. **C**. irEavas-positive cells from a mesenterial filament. **D**. Higher magnification views of the insets shown in **C**. The scale bars represent 50 µm in panels **A** and **C** and 20 µm in panels **B** and **D**. m, mesoglea; ect, ectoderm; end, endoderm.

Eavas was also found in the small number of cells of the tentacle endoderm (Fig. 8 A and B) and mesentery filament (Fig. 8 C and D).

### RT-PCR and Western blot analysis of *E. ancora* vasa gene products in unfertilized mature eggs

**Figure 9:**
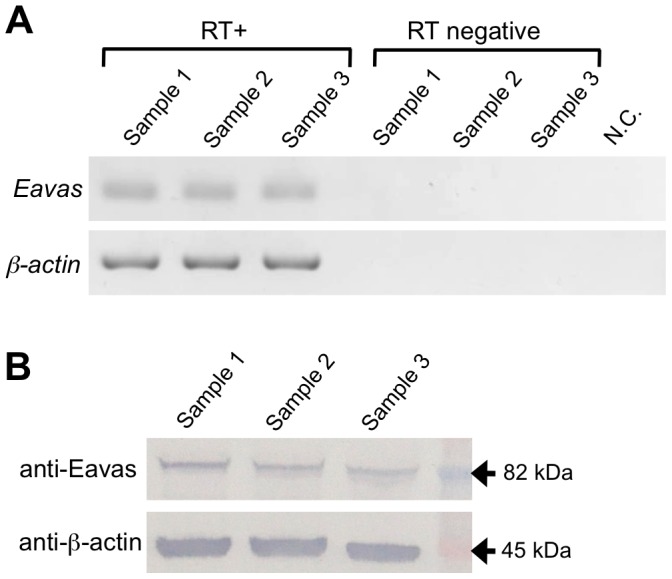
Expression of Eavas mRNA and protein in unfertilized mature eggs of *E. ancora*. **A**. RT-PCR analysis of *Eavas* expression in unfertilized mature eggs. The unfertilized mature eggs that were spawned in aquaria were collected from three different colonies, shown as Sample 1, Sample 2, and Sample 3. β-Actin was used as a positive control for the PCR reaction. Reactions lacking either the reverse transcriptase (RT negative) or template (N.C.) were included as negative controls for each set of reactions. **B**. Western blotting analysis of the anti-Eavas antibody. Protein extracts prepared from unfertilized mature eggs (12.5 µg) were separated by SDS-PAGE. After transferring to a nitrocellulose membrane, the proteins were immunoblotted with the anti-Eavas antibody (anti-Eavas) or anti-β-actin antibody (anti-β-actin). The unfertilized mature eggs that were spawned in aquaria were collected from three different colonies, shown as Sample 1, Sample 2, and Sample 3. The molecular weight markers are shown in the right.

To determine whether maternal *vasa* transcripts and proteins are present in unfertilized mature eggs, RT-PCR and Western blotting analysis were performed. RT-PCR of cDNAs from unfertilized mature eggs showed that the *Eavas* transcripts were present in unfertilized mature eggs. The control reactions lacking either the reverse transcriptase or template cDNA did not yield an amplified product (Fig. 9 A). Western blotting with the anti-Eavas antibody revealed an immunoreactive band of approximately 82 kDa (Fig. 9 B).

## Discussion

In the present study, we described the isolation of a full-length *vasa* cDNA and characterized the Vasa protein in corals. Sequence and phylogenetic analyses suggest that Eavas belongs to the *vasa* (and not the *PL10*) DEAD box helicase family [34]. In support of this argument, the anti-Eavas antibody we generated, which was raised against a polypeptide from Eavas that does not exist in *E. ancora* PL10 (EaPL10), demonstrated immunoreactivity against Eavas but not EaPL10. Immunohistochemical staining data revealed that Eavas is present in oogonia, early oocytes, developing oocytes, spermatogonia, and primary spermatocytes. Thus, Vasa is expressed in the germ cells of both sexes in the coral.

Between June and October for the males, and just after spawning in May for the females, Eavas immunoreactivity could be observed in unclustered cells with small nuclei and extended cytoplasms, which were located alongside the mesoglea between the mesenterial filaments and retractor muscles in both sexes. Because our histological analyses had suggested that these sites were the region of gametogenesis, we believe that the unclustered cells in these regions are most likely spermatogonia and oogonia or early oocytes. There are currently only a few papers that described the early germ cells of corals, i.e., unclustered spermatogonia and oogonia (also called interstitial cells), in details [1], [2], [35]. Thus, our data provide valuable new information for understanding early germ cell development in corals.

In most metazoans, the vasa genes are expressed exclusively in the germline. However, we found that vasa transcripts were produced not only in the coral mesentery, which is where the germ cells are located, but also in isolated tentacles. We also observed the Eavas protein in the small number of cells located in the tentacle endoderm and mesenterial filament. These findings are consistent with the observation that vasa family genes are expressed in both the somatic and germ cell lineages of several Cnidarian species [28]–[30] and Ctenophora [36]. The characteristics and cell types of Eavas positive cells in the tentacle endoderm and mesenterial filament are still unknown. Further histological analysis should be conducted in the future.

Coral germ cells are generally assumed to originate from interstitial cells [1], [2], [35], [37], [38], which can both self-renew and differentiate into multiple cell lineages, including the germline, as has been demonstrated in hyrdozoans [39]. However, it is still unclear whether corals possess true interstitial cells because the chemotherapy, cloning, and transplantation techniques alreadly being applied to characterize interstitial cells in hydrozoans [40]–[43] have not been used to identify interstitial cells in corals. Nevertheless, most corals can reproduce asexually by budding [44] and fragmentation [45], and it is quite probable that multipotent stem cells exist in the coral body. It has been reported that the *vasa* genes are expressed in the multipotent stem cells of planarian [46] and tunicate [47]. Also in hydra [29] and hydractinian [30] belonging to Cnidaria, *vasa* genes have been reported to express in the multipotent stem cells within interstitial cell population. The vasa described here may be useful as a marker protein for multipotent stem cells in corals.

Figure 6 demonstrated the features of Eavas protein that are distributed not only in the cytoplasm and perinuclear region of oocytes, but also within the nuclei of mature oocytes. Indeed, vasa is known as a translation factor, and its distribution have been exhibited in cytoplasm and perinuclear region but not in nuclei by immunohistochemistry using antibody against vasa in various animals [28], [30], [48]–[52]. Whatever the technique we used, immunofluoresence staining or alkaline phosphatase methods with NBT/BCIP colorization in immunodetection, Eavas signals consistently appeared within nuclei in mature oocytes. The signals in nuclei of mature oocytes are likely background signals caused by a non-specific binding of anti-Eavas antibody, though we cannot completely rule out the possibility of nuclear localization of Eavas. Figure 7 also documents an interesting feature of Eavas staining that a part of early male germ cells exhibited darker Eavas signals compared with other early male germ cells. The heterogeneity of the Eavas staining in the early spermatogonial population may be linked to the differences in certain characteristics, such as cell cycle phase, developmental stage or cell type.

In several metazoan species, such as *Drosophila*, *Xenopus*, and zebrafish, it was shown that the vasa gene product is maternally inherited [31]–[33]. Also, these maternally inherited vasa gene product is one of the germ cell determinants [31]–[33]. Our RT-PCR analysis of *Eavas* using unfertilized mature eggs proved that transcripts were present in the unfertilized mature eggs. Western blotting analysis demonstrated that Eavas proteins were also present in the unfertilized mature eggs, whereas immunohistochemical analysis against Eavas did not exhibit clear signals in the cytoplasm of mature oocytes as shown in Fig. 6. This inconsistency is probably caused by the dilution of Eavas protein with other various proteins accumulating within mature oocyte cytoplasm. Our findings raise the possibilities that Eavas products detected in the unfertilized mature could be the maternal origin, and vasa product would have a role in germ-cell determination in corals. Due to the inaccessibility to the fertilized embryo of *E.ancora*, we were unable to examine *Eavas* mRNA and its protein expression during embryogenesis. In starlet sea anemone *Nematostella vectensis* which belong to same class Anthozoa as corals, the mRNA distribution of two types of vasa, *Nvvas1* and *Nvvas2*, during embryogenesis have been reported, and suggested that both *Nvvas* genes appear to play roles in both somatic and germ line development [28]. Further analysis on mRNA and protein distribution of *vasa* during embryogenesis, and functional assay such as morpholino knockdown or RNAi would reveal the role of *vasa* gene in the mechanism of germ-cell determination and development in coral species.

In many metazoan species, gonadal somatic cells are known to play a crucial role in supporting the survival, proliferation, and differentiation of germline cells by enabling growth factors- and adhesion molecules-mediated intercellular communication [11], [12], [53], [54]. However, there is currently little known about the cells that support germ cell development in corals. Our histological analyses indicate that germ cell development in *E. ancora* usually occurs alongside and within the mesenterial mesoglea, between the retractor muscles and mesenterial filaments as in other coral species [1]–[10]. This finding raises the possibility that the somatic cells that appear at sites of gametogenesis could behave as supporting cells by fostering a favorable microenvironment for germ cells and producing growth factors. Future studies for the identification of the hormones and growth factors released from somatic cells and their receptors on germ cells will assist in clarifying the significance of supporting cells in coral reproduction. We believe that the anti-Eavas antibody generated here will be an invaluable tool in the classification of germ cells and somatic cells in coral tissues.

In conclusion, in this study, we described the annual gametogenic profiles of *E. ancora* by histological analysis with H & E staining. We also reported that the cloning of the coral germline marker vasa and production of a specific polyclonal anti-Vasa antibody. This study is the first to investigate early germ cell development in the scleractinian coral *E. ancora* using vasa as a germline marker. Thus, we believe that these studies shed light on the mechanism of germ cell determination and development in the early-evolved species.

## Materials and Methods

### Coral

*E. ancora* were collected at a depth of 10 m from Nanwan Bay, Kenting National Park near southern Taiwan (21°57′N, 120°46′E, see Fig. 1). Water temperatures of sampling sites were examined by a temperature/light data logger (Onset Computer Corporation, Cape Cod, MA; see Fig. 3 P). In order to understand when and where gametogenesis occurs in *E. ancora* coral bodies, thirteen colonies were labeled and collected over the course of the year on May 9, June 8, August 6, October 10, and December 5 of 2010 and January 10, February 14, March 15, April 14, and May 18 of 2011. The collection of the coral was approved by the administration office of the Kenting National Park in Nanwan. The experiments were conducted in accordance with the principles and procedures approved by the Institutional Animal Care and Use Committee, National Taiwan Ocean University, Taiwan.

### Histological analysis

The samples were fixed with 20% Zinc Formal-Fixx (Thermo Shandon, Pittsburgh, PA), decalcified with 5% formic acid (Panreac, Barcelona, Spain), and preserved in 70% ethanol. For histological analysis, a portion of each sample was embedded in paraplast plus (Sherwood Medical, St. Louis, MO), cut into 4 µm serial sections, and stained with haematoxylin and eosin Y (H & E staining, Thermo Shandon). For the *E. ancora* gametogenesis studies, more than 3 colonies of each sex were analyzed. Gametogenic stages 0-V were scored according to the criteria described in Table 1. For each stage of oogenesis, measurements of oocyte diameter were made using 30–782 randomly chosen oocytes that had visible nuclei. For the diameter calculations, diameter measurements were conducted along the longest dimension of the oocyte and then along a second axis passing perpendicularly through the first axis at the another longest dimension; the oocyte diameters presented are expressed as the average of the two measurements. All measurements were conducted using the Image J64 software (National Institutes of Health, Bethesda, MD). Spermatogenic stage determinations were made using approximately 50–250 germ-cell clusters. The frequency distribution percentages are given as the sum of the total percentages according to their frequency values. The diameters measurement for male germ cell clusters was conducted using approximately 100 randomly chosen clusters. For the diameter calculations, the same methodology used as oocyte diameter calculation was applied.

### RNA and protein extraction and cDNA synthesis

The frozen samples were homogenized in TRIzol reagent (Invitrogen, Carlsbad, CA) on ice, and total RNA and protein were extracted by following the manufacturer's protocol. First-strand cDNAs were synthesized from approximately 2 µg of total RNA using the SuperScript II reverse transcriptase (Invitrogen).

### Degenerate PCR cloning of the Eavas and EaPL10 cDNAs

To distinguish between the *E. ancora vasa* and *PL10* genes, degenerate PCR was used to obtain partial fragments of the *E. ancora vasa* (*Eavas*) and *PL10* (*EaPL10*) cDNAs. The degenerate primers (*Eavas* degFw1: 5′- GAGAACTKGCCTGTCAGATYTAC-3′; *Eavas* degRv1: 5′- GGTTGDGCCACAAGHAAATTGCA -3′) were designed against a region of *E. ancora vasa* that is highly conserved in the *vasa* homologues of other cnidarians (GenBank accession numbers: *Nematostella vectensis* AY730697; *Acropora digitifera* AB048853; and *Aurelia aurita* AB048858). The partial fragments obtained in the first reaction were extended in reactions containing one perfectly complementary and one degenerate PCR primer. The specific and degenerate primers (*Eavas* Fw1: 5′-TGCGGCAGCTGCAAAACAAAGGCTGCA-3′; *Eavas* degRv2: 5′-CGNGCNGCCACCGANGTRGC-3′) were designed against a region that is highly conserved in the *vasa* homologues of other invertebrates (GenBank accession numbers: *Ephydatia fluviatilis* AB047385; *Strongylocentrotus purpuratus* FJ605740; and *Crassostrea gigas* AY423380). The degenerate primers used to clone *EaPL10* (*EaPLl10* degFw1: 5′-TCTGGAAAAACTGCTGCCTTCC-3′; *EaPL10* degRv1: 5′-AGCCCAACACGTCCTCTGTCCA-3′; *EaPL10* nested degFw1: 5′-ACTGCTGCCTTCCTCATCCCAA-3′; *EaPL10* nested degRv1: 5′-AGCATATCCACAAGCCGTCCAG-3′) were designed using GenBank accession number AB048846 (*Acropora digitifera*). The PCR products were subcloned into the pGEM-T Easy vector (Promega, Madison, WI), and the inserted cDNA fragments were sequenced with the pUC/M13 forward and reverse primers using an ABI Prism 310 Genetic Analyzer (Applied Biosystems, CA).

### Cloning of the full length Eavas and EaPL10 cDNAs

To obtain the full length *Eavas* and *EaPL10* cDNA, 5′ and 3′ RACE were performed using the SMART™ RACE cDNA Amplification Kit (BD Biosciences Clontech, Franklin Lakes, NJ) and the following gene-specific amplification primers: *Eavas* 5′RACE 1st primer, 5′-GCTGGAACCAACATGGTGCTGTGGGAG-3′; *Eavas* 5′RACE nested primer, 5′- GGGAGAACAACTTGCGAGCTTC-3′; *Eavas* 3′RACE 1st primer, 5′-GGACGTTCCAGAGGATCAGAAGCGAGAC-3′; *Eavas* 3′RACE nested primer, 5′-GCTTAGCTGTTCAGGATCTGACAGGACCC-3′; *EaPL10* 5′RACE 1st primer, 5′-GCGTGGCTACCAGGAGATGACAGCCACG-3′; *EaPL10* 5′RACE nested primer, 5′-GCCGGGCATCAGGTGGGGGAGGAGGACC-3′; *EaPL10* 3′RACE 1st primer, 5′-GGTCCTCCTCCCCCACCTGATGCCCGGC-3′; *EaPL10* 3′RACE nested primer, 5′-CGTGGCTGTCATCTCCTGGTAGCCACGC-3′. Touchdown PCR was performed using the following amplification conditions: 94°C for 5 min; 5 cycles of 94°C for 30 s, 72°C for 2 min; 5 cycles of 94°C for 30 s, 69°C for 30 s, 72°C for 2 min; 33 cycles of 94°C for 30 s, 67°C for 30 s, 72°C for 2 min; followed by a final elongation step of 72°C for 7 min. The PCR products were resolved by electrophoresis, subcloned, and sequenced as described above.

### Sequence alignment and phylogenetic analysis

The deduced amino acid sequences of the cloned *Eavas* and *EaPL10* cDNAs were probed for homologous sequence entries using the National Center for Biotechnology Information website (http://www.ncbi.nlm.nih.gov/). For the phylogeny analysis, a subset of the Vasa/PL10 subfamily members from different species was retrieved from GenBank and phylogenetically compared with the *Eavas* and *EaPL10* clones obtained here. The region used for this analysis corresponds to the sequence AFLLPV…LDEA (from 276 to 385 residues of Eavas), where are available and comparable region among various taxa. The alignments were performed using MUSCLE and used to calculate a 1,000 bootstrap replicate phylogenetic tree (neighbor joining method) using MEGA 5 [55]. The cDNA sequences obtained in this study have been submitted to the NCBI nucleotide database (Eavas, JQ968407; EaPL10, JQ968406; β-actin, JQ968408).

### RT-PCR analysis of Eavas tissue distribution and unfertilized mature eggs

Tentacles that were fully extended in aquaria were cut using scissors. Samples of the mesentery, which included the gonadal regions, were isolated under a stereomicroscope (SZX16; Olympus, Tokyo, Japan). Unfertilized mature eggs that were spawned in aquaria were collected from three different colonies. RNA isolation and cDNA synthesis were performed as described above. The PCR conditions for measuring *Eavas* transcript levels were 94°C for 5 min; 35 cycles of 94°C for 30 s, 52°C for 30 s, 72°C for 30 s; followed by a final elongation step of 72°C for 7 min. The *Eavas*-specific PCR primers used were *Eavas* Fw: 5′-GATTGCAGAGAGTGCTGTAGGA-3′ and *Eavas* Rv: 5′-GAGACCAAGCTGAAGTGCCATT-3′. *E. ancora β-actin* was used as the internal control. The full length *β-actin* gene was obtained as a result of our transcriptome analysis. The *β-actin* cDNA was amplified under the following reaction conditions: 94°C for 5 min; 27 cycles of 94°C for 30 s, 52°C for 30 s, 72°C for 30 s; followed by a final elongation step of 72°C for 7 min. The *β-actin*-specific primers used were *β-actin* Fw: 5′-CACACCTTCTACAACGAACTCC-3′ and *β-actin* Rv: 5′-TGTAGGTGGTCTCATGGATACC-3′. Reactions in which the reverse transcriptase or template was omitted were used as negative controls for each primer set.

### Antibody production

A polyclonal anti-Eavas antibody was generated against the polypeptide C+AVGTSYGPAGGRFASRD, a sequence that is highly specific to Eavas and absent from EaPL10. The polypeptide was conjugated to ovalbumin (OVA) and used to immunize two rabbits. The rabbits were initially immunized with 400 µg of polypeptide conjugated with OVA, followed by 9 boosts of 500 µg at 2 weeks intervals. One rabbit serum exhibited strong immunoreactivity against peptide antigen assessed by dot-blot was collected on day 149. The antiserum was purified by an affinity column containing the 3 mg of peptide antigen (Yao-Hong Biotechnology Inc., Taipei, Taiwan). The purified antibody was validated by dot-blot (see Figure S1) and used in immunohistochemistry and Western blotting experiments.

### Immunohistochemistry

Immunohistochemical experiments were performed using the standard avidin-biotinylated-peroxidase complex (ABC) kit (Vector Laboratories, Burlingame, CA). Briefly, the hydrated sections were incubated for 10 min in 3% H_2_O_2_ and then for 1 h in 5% skim milk. The sections were immersed in the affinity purified anti-Eavas antibody (1∶2,000 in PBS, 1% skim milk) and incubated for 16 h at 4°C. After washing, the sections were incubated with a biotinylated goat anti-rabbit IgG antibody (Vector Laboratories; diluted 1∶2,000) for 30 min. Then, the sections were incubated in ABC solution and visualized using 3,3′-diaminobenzidine (DAB; Sigma-Aldrich). For the control samples, the anti-Eavas antibody was pre-adsorbed with 1 µg/ml of the peptide antigen. The sections were observed and photographed under a microscope (BX51; Olympus).

### Western blotting

The protein concentrations were determined by BCA protein assay kit (Pierce Biotechnology, Rockford, IL) by following the manufacture's protocol. The proteins were electrophoresed on NuPAGE Novex 10% Bis-Tris Mini Gels (Invitrogen) and transferred to a nitrocellulose membrane (Hybond-C Extra 0.45 µm; GE Healthcare UK Ltd., Amersham Place, Little Chalfont, Buckinghamshire HP7 9NA, England). After blocking, the membranes were incubated with the anti-Eavas antibody (1∶20,000 in TBS, 0.1% Tween 20 (TBST) with 1% skim milk) or anti-β-actin monoclonal antibody (Cat.# MAB1501; Millipore, Temecula, CA; 1∶10,000 in TBST with 1% skim milk) for 1 h at room temperature. The immunoreactive bands were detected after a 1-h incubation of the membranes in 0.25 µg/ml alkaline phosphatase-conjugated goat anti-rabbit IgG antibody (AnaSpec) diluted in TBST. Visualization was performed using the NBT/BCIP liquid substrate system (Sigma-Aldrich). As a control, the anti-Eavas antibody was pre-adsorbed with 200 ng/ml of the peptide antigen. The membranes were photographed with a digital camera (D90; Nikon, Tokyo, Japan).

### Statistics

Data are shown as the mean ± SEM. Statistical significance was analyzed using the Statistical Package for the Social Sciences (SPSS) with one-way analysis of variance (ANOVA) followed by Tukey 's test. Statistical significance level was set to *P*<0.05.

## Supporting Information

Figure S1 **Dot blot analysis of the immunoreactivity of the anti-Eavas antibody.** The synthetic antigen used for the antibody production was dotted on the nitrocellulose membrane (GE Healthcare) in the concentrations ranging from 100 to 0 ng. The antibody dilution factors were ranged from 1: 2,000 to 1: 16,000. A biotinylated goat anti-rabbit IgG antibody (Vector Laboratories; diluted 1∶2,000) was used as a secondary antibody. Dot blot analysis was performed using the standard avidin-biotinylated-peroxidase complex (ABC) kit (Vector Laboratories), and visualized using 3,3′-diaminobenzidine (DAB).
(TIFF)
