# Supplementary figures and images for: Germ Cell Development in the Scleractinian Coral Euphyllia ancora (Cnidaria, Anthozoa)
Source: PLoS One. 2012 Jul 27;7(7):e41569. doi: 10.1371/journal.pone.0041569 (PMC3407244; doi:10.1371/journal.pone.0041569)

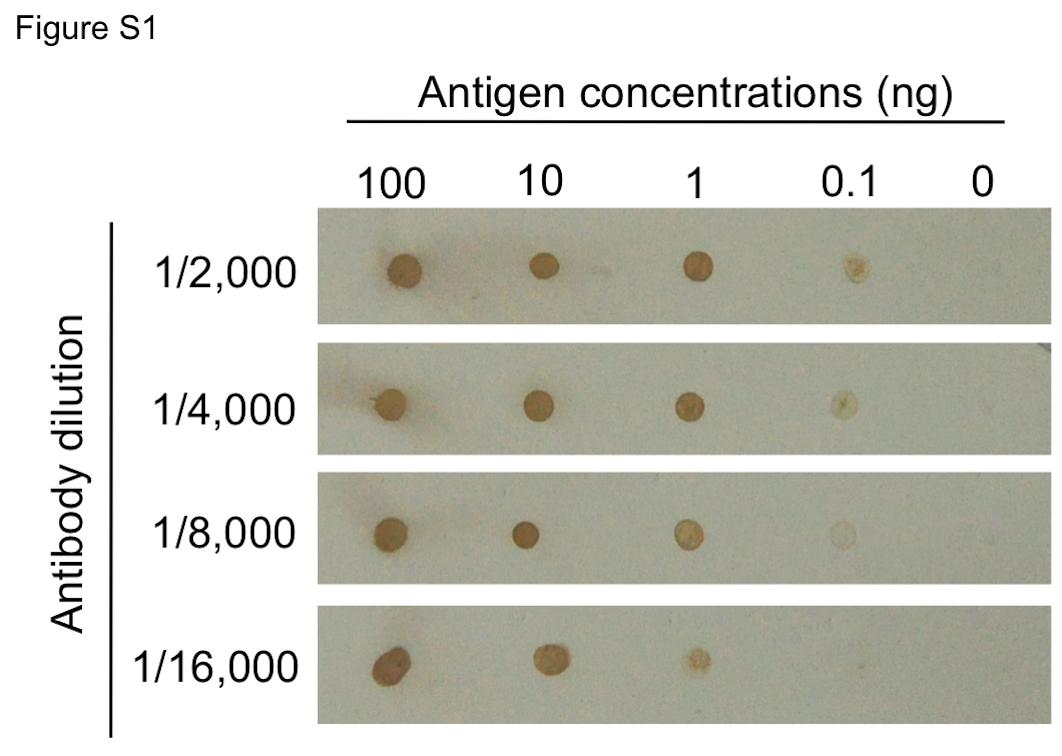

Supplement: Figure S1 — Dot blot analysis of the immunoreactivity of the anti-Eavas antibody. The synthetic antigen used for the antibody production was dotted on the nitrocellulose membrane (GE Healthcare) in the concentrations ranging from 100 to 0 ng. The antibody dilution factors were ranged from 1: 2,000 to 1: 16,000. A biotinylated goat anti-rabbit IgG antibody (Vector Laboratories; diluted 1∶2,000) was used as a secondary antibody. Dot blot analysis was performed using the standard avidin-biotinylated-peroxidase complex (ABC) kit (Vector Laboratories), and visualized using 3,3′-diaminobenzidine (DAB). (TIFF) [file pone.0041569.s001.tiff]
